# Editorial: Sleep and Mood Disorders

**DOI:** 10.3389/fpsyt.2019.00981

**Published:** 2020-01-16

**Authors:** Alexei Verkhratsky, Maiken Nedergaard, Luca Steardo, Baoman Li

**Affiliations:** ^1^ Laboratory Teaching Center, School of Forensic Medicine, China Medical University, Shenyang, China; ^2^ Faculty of Biology, Medicine and Health, The University of Manchester, Manchester, United Kingdom; ^3^ Achucarro Center for Neuroscience, IKERBASQUE, Basque Foundation for Science, Bilbao, Spain; ^4^ Center for Basic and Translational Neuroscience, Faculty of Health and Medical Sciences, University of Copenhagen, Copenhagen, Denmark; ^5^ Center for Translational Neuromedicine, University of Rochester Medical Center, Rochester, NY, United States; ^6^ Department of Physiology and Pharmacology “Vittorio Erspamer”, Sapienza University of Rome, Rome, Italy

**Keywords:** sleep disturbance, mood disorders, antidepressants, astrocytes, sleep–wake cycle

Sleep occupies almost one third of our life and it is necessary for survival of all species, including man. There is little doubt that sufficient, restorative sleep plays a critical role in maintaining physical and mental health. Evaluation of a good sleep always includes its quality, timing, quantity and rhythm. Sleep disturbances, including hypersomnia, insomnia, or irregular sleep patterns, result in various cognitive impairments and mood disorders (1, 2). Prolonged sleep deprivation or chronic sleep abnormalities are risk factors for the major depressive disorder (MDD) (3) and bipolar disorder (BD) (4), whereas disturbed sleep appears as a key symptom of mental diturbances (5, 6). Mechanisms connecting sleep deprivation and mood disorders remain, however, unclear.

The current Research Topic represents a collection of papers investigating the relationship between sleep abnormalities and mood disorders, as well as studies analyzing potential mechanisms connecting these pathologies. As described in the review by Steardo et al., the worldwide prevalence of sleep disorders is about 50% with even higher occurrence in psychiatric population. Sleep abnormalities are frequently associated with BD and are often a good predictor of a mood disorders. In this review, the alterations in the structure or duration of sleep are considered in all stages of BD. In particular the role of neuroglia in BD and the contributions of the different types of glial cells to BD and sleep abnormalities are discussed in depth. Specially, astrocytes are suggested to have an important role to the pathophysiology of BD through the loss of glial support and neuroprotection in disease-specific regions (7, 8). Furthermore, neuroglia cells are also reported to be a key target for the drugs used for the treatment of BD.

In the article by Li Y. et al., the relationship between stressful life events and sleep quality is discussed. The effects of stress on the sleep quality was investigated on more than 1,000 college students from a single province in China high correlation between scores of stressful life events and sleep quality has been reported. These results demonstrate that stressful life events can disturb the sleep quality of college students directly and indirectly, through rumination. The latter is defined as a response style that an individual tends to repeatedly think about the problematic situations being focused on negative emotions (9). Increasing psychological resilience could decrease both the direct and indirect effects stressful life events.

The influence of sleep problems in childhood and the relevant risk for mood disorders were further researched are in the focus of original paper by Wescott et al. This study is focused on the sleep parameters in offspring of parents with MDD or BD during both weeknights and weekends. Children of parents with MDD had longer sleep periods and total sleep time during weeknights than healthy controls. Thus, hypersomnia may be an early indicator of increased risk for depression in children and could be a relevant target for sleep-oriented interventions for individuals with high familial risk.

Effects of combined treatment with alprazolam and bailemian on the sleep quality of the patients after stroke were analyzed by Wang et al. The post-stroke insomnia is a very common symptom, albeit the treatment is often ignored (10). Alprazolam was discovered to improve the sleep efficiency and decrease the arousal times, rapid eye movement (REM) sleep and sleep latency, whereas bailemian significantly improved the sleep efficiency, total sleep time and the duration of N3. Moreover, the combination of these two drugs has synergistic qualities.

Besides the above three clinical reports, a novel approach for continuous recording the sleep–wake cycle in rodents is reported by Cui et al. Such a continuous sleep recording is required to reveal the sleep–wake, cycle which can reflect the brain maturational stages in postnatal rodent life (11). This paper reports a new method of continuous sleep recording involving two types of EEG electrodes, a milk-feeding system and temperature-controlled incubator. This technique allows characterization of sleep–wake cycle in the first postnatal month. This approach is suitable for recording continuous polysomnographic in infant rats and uncovers the ontogenetic features of sleep–wake cycle.

The sleep deprivation (SD) is known to aggravate various pathological processes including neuroinflammation. SD can induce the activation of nucleotide-binding domain and leucine-rich repeat protein-3 (NLRP3) inflammasome (12). The original article by Li X. et al., demonstrates that the activation of NLRP3 inflammasome is involved in the depressive-like behaviors induced by SD. Activation of NLRP3 inflammasome requires brain-derived neurotrophic factor (BDNF), which is decreased by SD. This study also shows that leptin augmented the anti-depressive effects of fluoxetine via increasing the expression of 5-HT_2B_ receptors in astrocytes. The decrease in BDNF by the activated NLRP3 inflammasome in astrocytes is the main pathological event of the depressive-like behaviors induced by SD, while the combination of fluoxetine and leptin can improve the therapeutic outcome for the depression triggered by SD.

The article by Lima et al. analyzed the consequences of the exposure of low- and high-exploratory rats during peri-adolescence; the authors evaluated and evaluated hippocampal immune-inflammatory/oxidative impairments as consequence of environmental stress (ES). The authors also analyzed the beneficial role of mood stabilizing drugs, lithium and valproate in prevention of ES-induced memory alterations. It appears that low- and high-exploratory rats exposed to ES present inflammatory phenotype, distinct anxiety-related behaviors and similar memory deficits, which all can be partially normalized by mood-stabilizing drugs.

The last two articles report potential pharmacological mechanisms of novel antidepressants, ketamine and vortioxetine. Ketamine, which is an ionotropic glutamatergic NMDA receptor antagonist, has been widely used as an anesthetic; recently however the rapid anti-depressant effect of ketamine treatment has been discovered. Rapid improvement of anhedonia and the depressive-like or anxious-like behaviors induced by the chronic unpredictable mild stress is described in the article of Wen et al. In the report by Cao et al., 100 patients with MDD were treated with vortioxetine, which is a multimodal antidepressant with multiple effects on neurotransmitter systems. It appears that vortioxetine significantly reduces anhedonia, which improves quality of life.

Taken together, this Research Topic covers several aspects of sleep disturbances and mood disorders and updates readers on the latest research in this field. These articles will provide researchers in many fields insights into which tools are available and thereby to further explore the relationship between sleep and mood disorders with the ultimate goal of developing effective therapeutic strategies.

## Author Contributions

Each of the authors have contributed intellectual content to the actual writing of the editorial.

## Funding

This study was supported by Grant No. 81871852 to BL from the National Natural Science Foundation of China, Grant No. XLYC1807137 to BL from LiaoNing Revitalization Talents Program.

## Conflict of Interest

The authors declare that the research was conducted in the absence of any commercial or financial relationships that could be construed as a potential conflict of interest.
